# Loss of miR-449a in ERG-associated prostate cancer promotes the invasive phenotype by inducing SIRT1

**DOI:** 10.18632/oncotarget.8061

**Published:** 2016-03-14

**Authors:** Parameet Kumar, Shashwat Sharad, Gyorgy Petrovics, Ahmed Mohamed, Albert Dobi, Taduru L. Sreenath, Shiv Srivastava, Roopa Biswas

**Affiliations:** ^1^ Department of Anatomy, Physiology and Genetics, Uniformed Services University of the Health Sciences, Bethesda, MD, 20814 USA; ^2^ Department of Surgery and Center for Prostate Disease Research, Uniformed Services University of the Health Sciences, Bethesda, MD, 20814 USA

**Keywords:** microRNA, miR-449a, SIRT1, ERG, prostate cancer

## Abstract

Epigenetic regulation by SIRT1, a multifaceted NAD^+^-dependent protein deacetylase, is one of the most common factors modulating cellular processes in a broad range of diseases, including prostate cancer (CaP). SIRT1 is over-expressed in CaP cells, however the associated mechanism is not well understood. To identify whether specific microRNAs might mediate this linkage, we have screened a miRNA library for differential expression in ERG-associated CaP tissues. Of 20 differentially and significantly expressed miRNAs that distinguish ERG-positive tumors from ERG-negative tumors, we find miR-449a is highly suppressed in ERG-positive tumors. We establish that SIRT1 is a direct target of miR-449a and is also induced by ERG in ERG-associated CaP. Our data suggest that attenuation of miR-449a promotes the invasive phenotype of the ERG-positive CaP in part by inducing the expression of SIRT1 in prostate cancer cells. Furthermore, we also find that suppression of SIRT1 results in a significant reduction in ERG expression in ERG-positive CaP cells, indicating a feed-back regulatory loop associated with ERG, miR-449a and SIRT1. We also report that ERG suppresses p53 acetylation perhaps through miR-449a-SIRT1 axis in CaP cells. Our findings provide new insight into the function of miRNAs in regulating ERG-associated CaP. Thus, miR-449a activation or SIRT1 suppression may represent new therapeutic opportunity for ERG-associated CaP.

## INTRODUCTION

Prostate Cancer (CaP) is the most common non-cutaneous form of cancer in men. It is also the second leading cause of cancer mortality in men in the USA. During the initiation and progression of prostate cancer, many genetic alterations occur. Oncogenic activation of the *ETS Related Gene* (*ERG*) as a result of gene fusions is the most common genomic alteration in prostate cancer reported to date [1-5]. ERG as an ETS related transcription factor has a milieu of transcriptional targets that regulate genes involved in various cellular processes including oncogenesis, inflammation, cell invasion, and DNA damage [1]. Moreover, inhibition of *ERG* in *TMPRSS2-ERG* expressing CaP cells (VCaP) suppresses the growth and invasiveness not only in VCaP cells but also in xenograft models [6]. ERG protein has been implicated as a key player in the progression from pre-invasive to invasive disease status of CaP [4, 5, 7]. Despite extensive investigations focused on understanding the molecular mechanisms and downstream mediators of molecular pathways that are important mediators of ERG-induced oncogenesis in CaP are continuously refined. This is essential for developing novel prognostic and therapeutic targets for CaP. Here we have examined the role of microRNAs in the development of ERG-associated CaP.

Recently, microRNAs (miRs, miRNAs) have emerged as important post-transcriptional regulators of gene expression. miRNAs are naturally occurring, highly conserved families of transcripts (∼22 nucleotides in length) that suppress expression of target genes via mRNA degradation and/or translational repression [8-12]. Dysregulation of miRNA expression has been identified in a number of cancers [13-20]. miRNAs can function as tumor suppressors or oncogenes and are important targets for development of anti-cancer therapeutics. Down-regulation or loss of function of a tumor-suppressing miRNA results in overexpression of target oncogenes. Conversely, activation or overexpression of an oncogenic miRNA results in the silencing of tumor-suppressing target genes. The discovery of miRNAs at previously identified chromosomal breakpoints, deletion and amplification sites in certain cancers implies their involvement in disease initiation and/or progression.

Global miRNA profiling in human cancer patient samples has identified a large set of miRNAs that are differentially expressed in cancer. In particular, several miRNAs such as miR-34, miR-145 and miR-31 have been shown to be down-regulated in CaP patients and regulate CaP progression through *c-Myc*, and *AR*. As miRNAs play important roles in gene regulation and it is plausible that loss of miRNAs may convey some of the ERG-induced prostate tumorigenesis. Although many studies have investigated the downstream genes of ERG, very few studies have examined the miRNAs that are regulated by ERG or regulate ERG. miR-30 tumor suppressor has been demonstrated to connect the major EGF/Src signaling to ERG providing mechanistic insights into EMT features of ERG-positive CaP cells [21]. miR-145 has been shown to inhibit ERG expression by directly targeting its 3′-UTR [22]. Thus, loss of miR-145 may provide a *TMPRSS2–ERG* gene fusion-independent means to ERG up-regulation in CaP. It has been demonstrated that miR-221 is down-regulated in CaP patients [23]. A recent study has implicated miR-200c in ERG-associated CaP [24]. However, a comprehensive analysis of miRs in relation to ERG status is lacking in CaP tissues.

Here we report that loss of miR-449a and a subsequent induction in SIRT1 expression causes the invasive phenotype of ERG-positive CaP. We have analyzed the miRNA signature in RNA samples obtained from Laser Capture Micro-dissected (LCM) epithelial cells of ERG-associated CaP tissues of patients undergoing radical prostatectomy, including ERG-positive as well as ERG-negative CaP. Our analyses indicate that indeed an ERG-regulated miRNA program exists in CaP tissues that can distinguish between ERG-positive and ERG-negative CaP tumors. We have thus identified the loss of expression of miR-449a in ERG-positive CaP compared to ERG-negative CaP tissues. Our data indicate that over-expression of miR-449a in a cell culture model can rescue the disease phenotype of ERG-positive CaP, including cell migration, anchorage-dependent growth and cell invasiveness. The mechanism appears to be mediated by up-regulation of SIRT1, which belongs to the Sir2 (silent information regulator 2) family of sirtuin class III histone deacetylases, which we have identified as a direct target of miR-449a. Furthermore, we also find that suppression of SIRT1 induces significant reduction in ERG expression in ERG-positive CaP cells. These data suggest an interesting feed-back regulatory loop associated with ERG, miR-449a and SIRT1; whereby increased expression of ERG suppresses miR-449a and up-regulation of its target gene SIRT1, which in turn enhances expression of ERG. Moreover, we find that ERG suppresses p53 acetylation, which is perhaps mediated through miR-449a-SIRT1 axis in CaP cells. These mechanisms are potential therapeutic targets for ERG-associated CaP.

## RESULTS

### Prostate cancer cells express ERG-specific miRNAs

A number of miRNAs have been shown to influence key cellular processes involved in prostate tumorigenesis. However, studies of miRNAs in the context of *TMPRESS2-ERG* gene fusion are limited. Here, we have determined a comprehensive miRNA expression profile in ERG-positive, and ERG-negative CaP tumor tissue. We have identified ERG-associated specific microRNAs in CaP tissues (Table 1). Out of the analyzed 365 miRNAs, 20 were significantly different in the ERG-positive CaP tissues and ERG-negative CaP tissues (n=6, Table 1). We identified miRNAs for which the fold difference was at least *ca*. 50%, and the p value for the difference was <0.05. Of the 20 differentially expressed miRNAs, four were elevated, and 16 were reduced in the ERG-positive CaP tissues. The complete list of microRNA expressions in CaP tissues, as analyzed by Taqman human miRNA arrays, is included in Supplementary Table 1. Statistical analyses of the data were performed with STATMINER software (Integromics, Inc.).

**Table 1:** miRNA expression levels in ERG-positive human CaP tissues relative to ERG-negative CaP tissues

microRNAs	RQ	p-value	Expression
*hsa-miR-874	−22.6	0.00123	↑
hsa-miR-520g	−21.7	0.00275	↑
hsa-miR-125a-3p	−5.1	0.00740	↑
hsa-miR-129-5p	−14.2	0.03447	↑
*hsa-miR-449a	7.7	0.001218	↓
hsa-miR-589	5.2	0.00222	↓
hsa-miR-532-5p	6.4	0.01501	↓
hsa-miR-370	7.1	0.01860	↓
hsa-miR-520f	4.3	0.02457	↓
hsa-miR-886-5p	3.7	0.02709	↓
hsa-miR-331-5p	2.7	0.02821	↓
hsa-miR-382	7.7	0.02926	↓
hsa-miR-149	5.2	0.03480	↓
hsa-miR-139-3p	5.5	0.03996	↓
hsa-miR-362-5p	3.5	0.04086	↓
hsa-miR-140-3p	2.9	0.04167	↓
hsa-miR-539	10.6	0.04207	↓
hsa-miR-872	12.6	0.04285	↓
hsa-miR-576-3p	7.1	0.04468	↓
hsa-miR-660	4.8	0.04575	↓

The miRNAs which exhibit altered expression, analyzed by Taqman qPCR miRNA arrays, are listed with respective relative quantification in the ERG-positive compared to ERG-negative human CaP tissues: the miRNAs are ordered on the basis of significance. Four miRNAs that are up-regulated (↑) and the 16 that are down-regulated (↓) are indicated (p<0.05). The two most significantly altered microRNAs, miR-874 (↑) and miR-449a (↓) are indicated by asterisk (*). RQ: Relative Quantitation.

**Figure 1:**
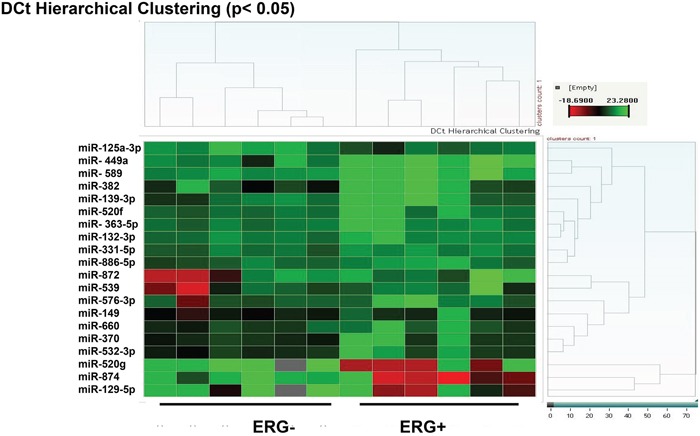
miRNA expression in ERG-associated CaP tissues Epithelial cells were isolated from human CaP tissues by Laser Capture Micro-dissection. The miRNA expression profile in ERG-positive CaP cells compared to that in ERG-negative CaP cells shows significant differences (p value ≤ 0.05, n = 6) in the expression of 20 miRNAs (Red: high-expression, Green: low-expression).

Consistently, these differentially expressed miRNAs appear to contribute to a composite ERG-associated CaP microRNA signature. When all 20 microRNAs are compared using a hierarchical cluster algorithm, the dendrogram clearly distinguishes between six independent samples with ERG-positive CaP and ERG-negative CaP (Figure 1). The initial analyses indicate that indeed an ERG-regulated miRNA program exists, which is comprised of 20 miRs (Figure 1). These include over-expressed miRs (viz. miR-874, miR-129-5p, miR-125a-3p, and miR-520g) and down-regulated miRs (viz. miR-449a, and miR-660) in CaP tissues. These miRNAs are potential biomarkers for CaP stratified by ERG status. The data in Table 1, ordered by significance, indicate that the miRNAs with the most significant differential expression are miR-449a (down-regulated) and miR-874 (up-regulated).

### Validation of ERG-associated miRs in prostate cancer cells

**Figure 2:**
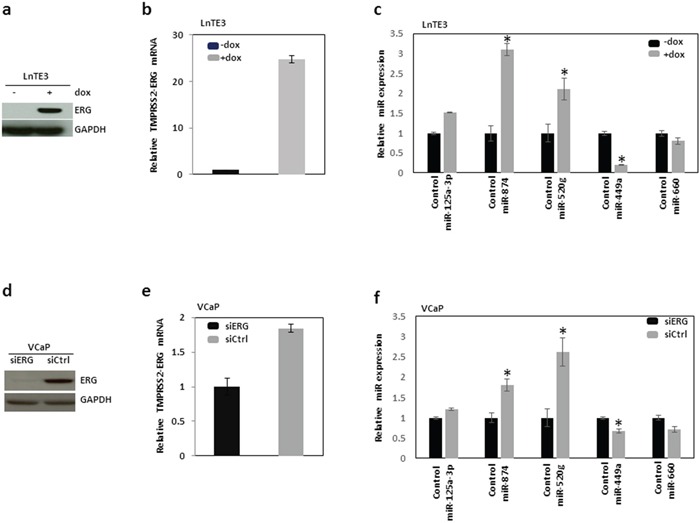
Validation of ERG-associated miRNAs in CaP cells The expression of a selected set of miRNAs, which exhibited altered expression in ERG-positive CaP tumor tissues compared to ERG-negative CaP tumors is further validated in two different CaP cell lines, LnTE3 and VCaP cells. LnTE3 cells (LNCaP-lentivirus TMPRESS2:ERG3, inducible) were treated with doxycycline (1μg/ml) for 72 hrs to induce ERG expression: **a.** protein analyzed by immunoblot and **b.** TMPRESS2-ERG mRNA analyzed by qPCR. **c.** The expression of miR-125-5p, miR-520g, miR-874, miR-449a and miR-660 was analyzed by Taqman miR-specific assays in LnTE3 cells. VCaP cells were treated with siERG or control siRNA, both **d.** ERG protein and **e.** mRNA were analyzed by immunoblot and qPCR, respectively. **f.** The expression of miR-125-5p, miR-520g, miR-874, miR-449a and miR-660 was similarly analyzed by miR-specific Taqman assays in VCaP cells. For all miR-specific assays RNU48 was used as an endogenous control. The data reflect averages of at least three independent experiments (* indicates p<0.05).

Next we performed an independent validation of significantly altered miRNAs in ERG-positive CaP compared to ERG-negative CaP tissues, using Taqman-based miR-specific assays. We analyzed the expression of a subset of miRNAs in VCaP cell line harboring *TMPRSS2-ERG* fusion [3, 6]. Additionally, we also employed LNCaP cells with doxycycline inducible *ERG* named LnTE3 cells (LNCaP-lentivirus *TMPRESS2:ERG3*, inducible) for similar analyses. As depicted in Figures 2a and 2b, LnTE3 cells exhibits increased expression of ERG after doxycycline treatment.

Consistent with the miR expression data in CaP tissues, we observe a similar trend in expression of selected subsets of miRNAs in LnTE3 cells (Figure 2c). To further validate the specificity of ERG on the regulation of miRNAs, we performed similar experiments in VCaP cells, harboring endogenous high levels of ERG. VCaP cells in which ERG was depleted via siRNA-mediated knock-down (Figure 2d and 2e) were also included as ERG-negative controls. As observed in CaP tissues and in ERG inducible LnTE3 cells, similar trends were observed for selected miRNA expression (i.e. miR-874, miR-520g and miR-125-5p up-regulated and miR-449a and miR-660 down regulated) in VCaP cells (Figure 2f).

### Effect of miR-449a and miR-874 on ERG-associated prostate cancer-specific genes

**Figure 3:**
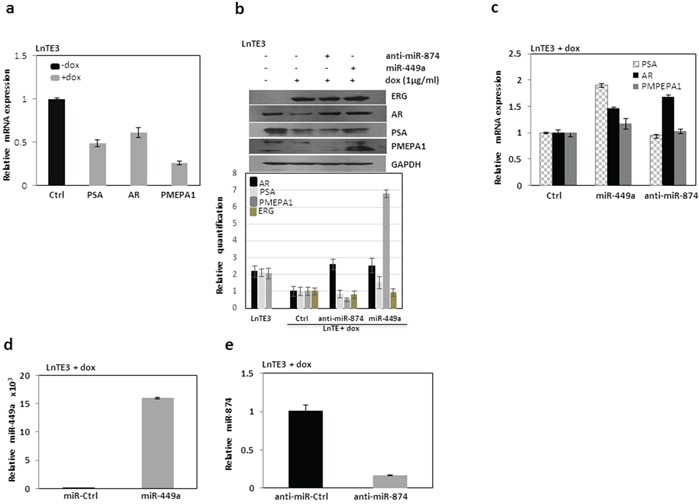
Analyses of ERG-associated genes in LnTE3 **a.** The expression of ERG-associated genes including *AR, PSA/KLK3 and PMEPA1* were analyzed by qPCR using TaqMan gene expression assays in LnTE3 cells (2.5 × 10^5^) induced with doxycycline (dox, 1μg/ml). **b.** AR, PSA and PMEPA1 protein expression was analyzed by immunoblot in LnTE3 cells: Lane 1 (control) without dox, Lane 2 with dox, Lane 3 with dox and anti-miR-874 (20 nM) and Lane 4 with dox and pre-miR-449a (20nM). The relative quantification is indicated below. **c.** Corresponding mRNA expression was analyzed by qPCR. The modulation of miR-449a and miR-874 in LnTE3 cells was also separately verified as described. **d.** miR-449a expression was assayed in dox-treated LnTE3 cells incubated with pre-miR-449a (miR-449a, 20nM) and its negative control (miR-ctrl, 20nM) for 48 hr miR-specific TaqMan assay. **e.** Similarly, miR-874 expression was analyzed by TaqMan assays in dox-treated LnTE3 cells incubated with anti-miR-874 (20 nM) or respective negative control (anti-miR ctrl, 20nM) for 48hr.

Next, we selected the top two most significant candidate miRNAs, miR-449a (down-regulated) and miR-874 (up-regulated) (Figure 1 and Table 1) for functional analyses. Since ERG is known to interfere with AR-signaling [6, 25, 26], we analyzed the expression of AR and AR-regulated genes in LnTE3 CaP cell line. ERG over-expression causes a suppression of *AR* as well as its target mRNAs and their respective protein expression including PSA and PMEPA1 in LnTE3 cells (Figures 3a and 3b). We also observed similar responses in VCaP cells (Supplementary Figures 1a and 1b).

Subsequently, we analyzed the effect of modulation of these two miRNAs (Figures 3d and 3e) on AR and its target genes in ERG inducible LnTE3 cells. Our data indicate that over-expression of miR-449a increased the expression of *AR* and its downstream target *PMEPA1*, compared to controls in ERG inducible LnTE3 cells, both at the transcript and protein levels (Figures 3b and 3c). Concurrently, we also suppressed the expression of miR-874 using anti miR-874 in the LnTE3 cell lines and similarly analyzed the expression of *AR* and its target genes. As depicted in Figure 3b and 3c, suppression of miR-874 increased AR protein expression, while no significant changes were observed in the expression of PMEPA1 and PSA proteins. However, the corresponding transcript levels of these genes were unaffected except for *AR*. These results indicate that overexpression of miR-449a have marked effect on AR and its target genes, while suppression of miR-874 only affects AR expression. Similar observations are also reported for VCaP cells (Supplementary Figures 1b and 1c).

### Effect of miR-449a and miR-874 on the phenotype of the ERG-associated prostate cancer cells

**Figure 4:**
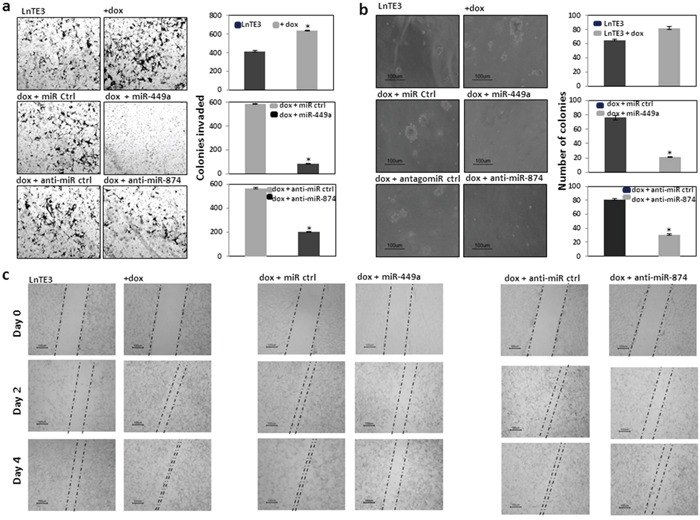
Functional effect of restoring miR-449a and miR-874 in CaP cells LnTE3 cells (2.5 × 10^5^) were seeded in 6-well plates and were either untreated, or treated with doxycycline (1μg/ml) and transfected with pre-miR-449a or anti-miR-874 and respective controls the following day. **a.** For analyses of cell invasion, an aliquot of transfected cells (1 × 10^5^) were suspended in serum-free medium and incubated in transwell Matrigel invasion chambers for 48 h. The invaded cells were then stained with crystal violet and photographed. ERG over expression in LnTE3 cells enhance cell invasion as shown (top panel). Cell invasion in ERG-induced LnTE3 cells exhibiting increased miR-449a expression (middle panel) or those in which miR-874 was suppressed (bottom panel) are indicated. Experiments were repeated at least 3 times and one representative result is shown. Quantitative graphical presentation of representative results of cell invasion assays is shown for each category on the right side. Values shown are the average of at least three independent experiments (means ± SD). *p<0.05 **b.** Anchorage-independent growth was determined by the Colony formation assay in soft agar. LnTE3 cells were transfected as mentioned above and 24 hr post transfection, 1 × 10^5^ cells were mixed with 2 ml of culture media containing 0.3% Difco agar Noble and then overlaid on 1 ml of 0.5% agar in 6 well plate culture dishes. After 3-4 weeks, colonies were visualized under microscope at a magnification of 10X and counted. Representative cell colonies in soft agar are shown. Induction of ERG enhanced formation of soft agar colonies by number and size as apparent after 21 days (top panel). Overexpression of miR-449a or suppression of miR-874 reverts the effects as depicted (middle and bottom panel, respectively). Quantitative analysis of colony numbers is shown in the graphical representation for each comparison. Values shown are the average of at least three independent experiments (means ± SD). *p<0.05. **c.** For wound scratch assay, LnTE3 cells were seeded in 6 wells plate with or without doxycycline and grown up to 90% confluence. A single wound was made in center of cell monolayer. Cells were washed 2 times with PBS and transfected with respective microRNAs or controls. The wound closure areas were visualized under an inverted microscope as shown. The ERG overexpression increased cell migration (left panel), which was inverted by overexpression of miR-449a (middle panel) or suppression of miR-874 (right panel). Scratch assays were performed at least three times and representative results are shown.

Cell migration and invasion are fundamental functions underlying cellular processes, including cancer metastasis [27]. ERG has been shown to induce tumorigenesis by increasing cell invasion [3, 28]. Therefore, we first determined whether ERG affects the invasive phenotype of these cells, using Matrigel assays. As shown in Figure 4a (top panel), ERG over-expression significantly increased the invasiveness of CaP cells (∼30%). We further analyzed the role of miR-449a and miR-874 in promoting the invasive phenotype of LnTE3 cells. Thus we modulated these miRNAs in ERG inducible LnTE3 cells, by over-expressing miR-449a or depleting miR-874. Our data indicate that increased expression of miR-449a in LnTE3 cells suppresses the invasive phenotype by ∼80% (Figure 4a, middle panel) as compared to its control. Additionally, inhibition of miR-874 expression also causes ∼40% reduction in the CaP cell invasiveness (Figure 4a, bottom panel), compared to scrambled controls. The data representative of three independent experiments is depicted in adjacent panels.

The ability of the cancer cells to adhere and grow by anchorage-independent mechanisms is very important to determine their potency to evade cell death and finally metastasize. In the presence of ERG, a striking increase in soft agar colony formation was observed. ERG-positive LnTE3 cells formed larger, disseminated, as well as greater number of colonies compared to smaller, smooth edged colonies formed by the control cells, after 21 days (Figure 4b, top panel). Conversely, the soft agar colony formation assay demonstrated that the anchorage-independent growth potential of the miR-449a and miR-874 modulated cells were highly hindered compared to the control transfected cells after 21 days (Figure 4b, middle and bottom panel). This effect is more prominent with over expression of miR-449a. The data representative of three independent experiments are included in the adjacent panel of each experimental group (* indicates p<0.05).

In a wound-healing migration assay, we observed that the speed of wound closure was significantly increased with over expression of ERG in LnTE3 cells (Figure 4c). In parallel, we modulated the expression of miR-449a and miR-874. Modulations of these miRNAs decreased cell migration (Figure 4c). This effect is more significant with miR-449a suggesting that miR-449a has an inhibitory effect on cell migration in ERG-associated CaP.

In summary, the phenotypic rescue of LnTE3 cells is more effective with increased expression of miR-449a than with suppression of miR-874. Thus, in the subsequent experiments we focused on analyzing the mechanism by which miR-449a affects CaP disease phenotype.

### Identification and validation of SIRT1 as novel ERG-associated prostate cancer-specific target of miR-449a

**Figure 5:**
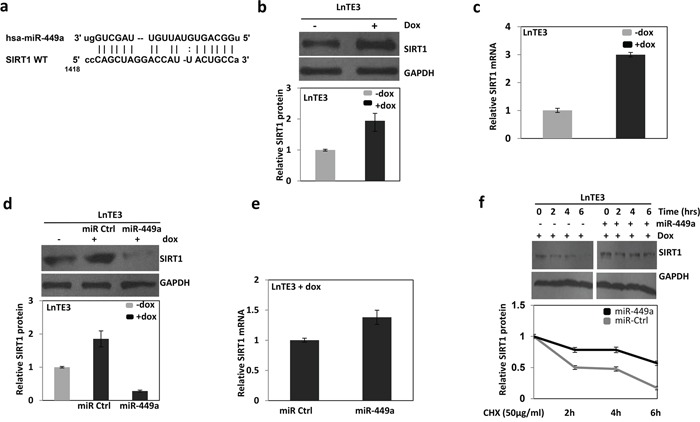
ERG and miR-449a reciprocally regulate the expression of SIRT1 in ERG-associated LnTE3 cells **a.** The SIRT1 3′UTR miR-449a putative target site. SIRT1 expression in LnTE3, either untreated or treated with doxycycline (1μg/ml) was analyzed by **b.** immunoblot and **c.** qPCR. Similarly, SIRT1 **d.** protein and **e.** corresponding mRNA was also analyzed in LnTE3 cells treated with doxycycline and transfected with pre miR-449a as well as respective controls. The graph below the immunoblot represents relative protein quantification. **f.** Dox-treated LnTE3 cells transfected with pre miR-449a or respective control (20nM each) for 48 hr. Subsequently, the cells were incubated with cycloheximide (CHX, 50μg/ml) for the indicated time intervals (0, 2, 4 and 6 hours) and SIRT1 expression was analyzed by immunoblot. The relative amount of SIRT1 protein was quantified (Bottom panel). Error bars represent data from three independent experiments (mean ± SD).

To find potential targets of miR-449a, we used online search tools (*viz*. Target scan, mirwalk, PicTar, miRanda). Among many predicted targets, miR-449a is predicted to target *SIRT1* mRNA (Figure 5a). We first analyzed the expression of SIRT1 in ERG inducible LnTE3 cells. ERG over-expression significantly increases SIRT1 expression, both at protein (Figure 5b) and mRNA (Figure 5c) levels, as measured by immunoblot and qPCR, respectively. Similarly, we also observed high expression of SIRT1 in ERG-positive VCaP cells (Supplementary Figure 2). We further analyzed the transcript and protein expression of SIRT1 in LnTE3 cells transfected with scrambled control or pre-miR-449a. Over-expression of miR-449a in LnTE3 cells suppresses SIRT1 protein, without affecting the corresponding *SIRT1* transcript (Figure 5d and 5e), compared to miR-control-treated LnTE3 cells. Collectively, these data suggest that miR-449a reduce SIRT1 expression by inhibiting translation of SIRT1 without affecting mRNA levels.

Since miR-449a appears to cause translational suppression of SIRT1, we further analyzed the effect of miR-449a on the stability of SIRT1 protein. ERG inducible LnTE3 cells were transfected with pre-miR-449a or a control miR. Subsequently, we inhibited protein synthesis with cycloheximide (CHX) for the indicated time intervals of 2, 4 or 6 hours. The corresponding protein level of SIRT1 was analyzed by immunoblot. Indeed, over-expression of miR-449a reduced the rate of degradation of SIRT1 protein as compared to controls (Figure 5f). The relative amount of SIRT1 protein was quantified (ImageJ software) as indicated in Figure 5f. Our result demonstrates that miR-449a increases the stability of SIRT1 protein. The data is representative of three independent experiments (mean ± SD).

**Figure 6:**
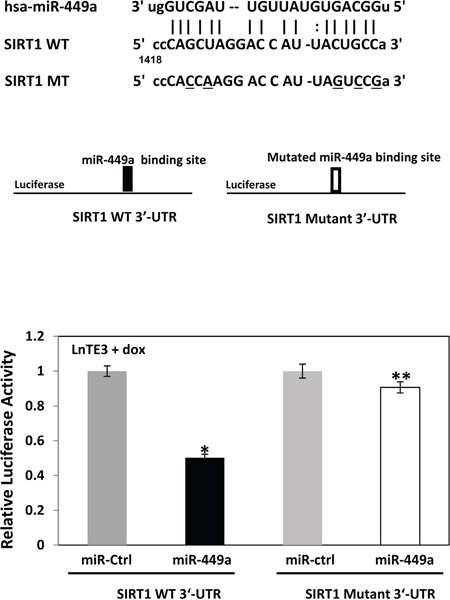
SIRT1 is a direct target of miR-449a in ERG-associated LnTE3 cells The miR-449a target 3′-UTR SIRT1 sequences as well as that of the mutated-derivative are indicated. Luciferase reporter assays were performed in ERG-inducible LnTE3 cells transfected with pMIR-Report vectors, either controls or those containing SIRT1 3′-UTR target sequences of miR-449a (both wildtype [WT] and mutant [mt]), in the presence or absence of pre-miR-449a. The data reflect averages of at least three independent experiments (* indicates p<0.05, ** indicates p>0.05).

To confirm SIRT1 as a direct miR-449a target, we cloned 3′-UTR predicted target sequence of SIRT1 (as shown in Figure 5a) downstream to a luciferase reporter gene in the pMIR-Report vector. This vector has a luciferase reporter gene under the control of a mammalian promoter/terminator system, with a miR-target cloning region downstream of the luciferase translation sequence. Luciferase activity will be reduced or abolished if the 3′-UTR sequence is a valid miRNA target. A control pMIR-Report β-gal vector is simultaneously transfected to normalize the transfection efficiency. Also, pMIR-Report-derivatives in which the SIRT1 mRNA 3′-UTR target sequences have been mutated has been included as controls for these assays (Figure 6). The vectors (WT or mutant or control) were transfected into ERG inducible LnTE3 cells incubated with pre-miR-449a and its scrambled control. Luciferase activity is reduced (∼60%) when WT SIRT1 target site for miR-449a is used. In contrast, reporter harboring the mutant miR-449a target sequence showed unaltered luciferase activity. Thus SIRT1 is indeed a direct target of miR-449a in ERG-associated CaP cells.

### SIRT1 regulates invasive cancer phenotypes of ERG-associated prostate cancer cells

**Figure 7:**
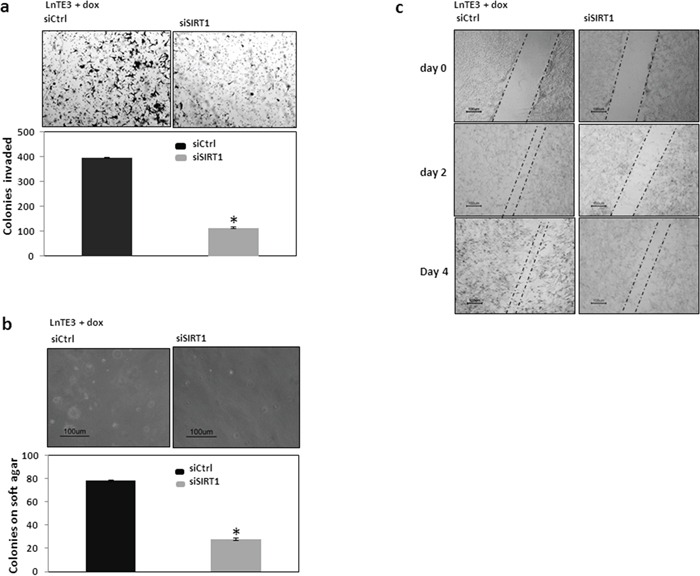
Functional analyses of SIRT1 in ERG-inducible LnTE3 cells LnTE3 cells were treated with doxycycline (1μg/ml) and transfected with siSIRT1 and its controls (20nM each) using siPORT transfection reagent the following day. **a.** Cell invasion **b.** Colony formation and **c.** Cell migration at different time intervals were analyzed as described earlier. The data is representative of three independent experiments (mean ± SD).

Migration and invasion are key processes that facilitate cancer progression and *SIRT1* is known to increase these processes. Since, *SIRT1* is one of the direct targets of miR-449a, we sought to determine if the suppression of *SIRT1* could attenuate these processes, independent of the effect that miR-449a may have on other transcripts. As shown in Figure 7a, siSIRT1-mediated knockdown of *SIRT1* inhibits invasiveness in ERG-positive LnTE3 cells as compared to control siRNA-treated cells. The data shown in the figure is representative of three independent experiments (mean ± SD).

To test the possibility that SIRT1 could affect the anchorage-independent growth of LnTE3 cells, we suppressed SIRT1 expression and examined the ability to form colonies in soft agar. As shown in Figure 7b, suppression of SIRT1 induces a significant suppression in colony formation on soft agar. The corresponding graphical representation of three independent experiments is also depicted.

In a wound-healing migration assay, we had observed that the speed of wound closure was significantly decreased in ERG-positive LnTE3 cells with increased miR-449a (Figure 4a), suggesting that miR-449a has an anti-migratory effect. Consistent with previous findings, in this study we observe that siRNA-mediated depletion of SIRT1 in ERG-positive LnTE3 cells also suppressed cell migration (Figure 7c).

These data strongly support the role of SIRT1 proteins in mediating the functional effects of miR-449a in ERG-associated CaP.

### ERG suppresses p53 acetylation through miR-449a-SIRT1 axis

**Figure 8:**
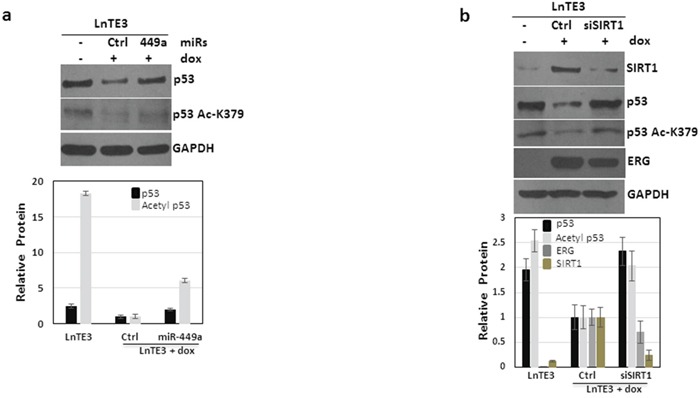
ERG regulates p53 signaling LnTE3 cells (2.5 × 10^5^) were either untreated or treated with doxycycline (1μg/ml) and transfected with pre miR-449a or siSIRT1 RNA and respective controls (20nM each) using siPORT transfection reagent on the following day. **a.** Total and acetylated p53 proteins levels were analyzed by immunoblot in dox-treated LnTE3 cells incubated with pre miR-449a (48 h). **b.** SIRT1, ERG, p53 (total and acetylated) protein expression was analyzed by immunoblot in dox-treated LnTE3 cells incubated with siSIRT (48 h). The relative quantification are indicated below the corresponding immunoblot. The data is representative of three independent experiments (mean ± SD).

It is well established that SIRT1, a NAD+ dependent histone deacetylase, affects the acetylation of p53 [29]. We observe that increased expression of ERG induces a reduction in total p53 protein (Figure 8a and Supplementary Figure 2). Subsequently we analyzed the functional expression of p53 in ERG-associated cells. Indeed, our data indicate that in addition to suppressing total p53 expression, ERG also suppresses p53 acetylation (Figure 8a). Next we analyzed the effect of modulation of miR-449a and SIRT1 on p53 acetylation. As depicted in Figures 8a and 8b, we find that suppression of SIRT1, either directly by siSIRT1 or via over-expression of miR-449a, increases total p53 expression as well as p53 acetylation. Thus, our data demonstrate that ERG suppresses acetylation of p53 via suppression of miR-449a and a consequential increase in SIRT1. Furthermore, the expression of ERG was significantly reduced (∼40%) by depletion of SIRT1 in ERG-associated CaP cells.

## DISCUSSION

miRNAs are known to be important regulators of biological process in variety of diseases including cancer. Despite recent studies targeted towards understanding the role of microRNAs in the development of ERG-associated CaP, a comprehensive analysis is lacking. Therefore we have examined the miRNA signature in RNA samples of ERG-associated CaP tissues of patients undergoing radical prostatectomy. Indeed an ERG-regulated miRNA program exists in CaP tissues that can distinguish between ERG-positive and ERG-negative CaP tumors. The miRs that exhibit significantly altered expression and distinguish between ERG-positive and ERG-negative include over-expressed miRs (viz. miR-874, miR-125a-3p, miR-129-5p, and miR-520g) and down-regulated miRs (viz. miR-449a, miR-589 and miR-660) in CaP tissues (n=6, p<0.05). These miRNAs may be potential candidate prognostic biomarkers for ERG-positive CaP. Consistent with our findings, previous reports indicate that these miRs are associated with various type of cancer where their altered expression renders them as onco-miRs or tumor suppressors. miR-520g is up regulated in hepatocellular carcinoma and induces epithelial mesenchymal transition [30]. miR-125a is associated with inhibition of EMT in cancer [31]. Of these significantly altered microRNAs, miR-449a exhibits maximum down-regulation while miR-874 is the most up-regulated miR in ERG-positive CaP tissues compared to ERG-negative CaP tissues. Consistently, similar trend in expression of these two miRs was also observed in a cell culture model of ERG-inducible LNCaP and VCaP cells.

Here we focused on the top candidate miRs, miR-449a which is most significantly suppressed and miR-874 which is profoundly up-regulated in ERG-positive CaP tissues compared to ERG-negative CaP. We find that both miR-449a and miR-874 are associated with the regulation of *ERG* related genes, *AR*, *PSA/KLK3* and *PMEPA1*. One of the most important roles of ERG that has been shown in various studies is its ability to increase cell migration and invasion. To study the role of these microRNAs in regulating these functions, we examined the cell invasion, cell migration and colony formation assays in LnTE3 cells. Phenotypic biological assays indicate that restoring the expression of miR-449a (by over-expression) or miR-874 (by suppression) can rescue the invasive cancer phenotype of the ERG-associated CaP cells. Recent studies have implicated miR-874 to be a positive regulator of cancer networks [32]. However, miR functions are disease specific and in the CaP tissues analyzed, we observe significant up-regulation of miR-874.

miR-449a is one of the best studied miRNAs in cancer biology. Extensive research has shown that miR-449a functions as a tumor suppressor in variety of cancers including endometrial cancer [33], gastric adenocarcinoma [34], retinoblastoma [35], neuroblastoma [36] and bladder cancer [37] by reducing cell proliferation, migration and invasion. Recent reports indicate that miR-449a also suppresses the epithelial-mesenchymal transition and metastasis of hepatocellular carcinoma [38]. Surprisingly, very few papers have explored miR-449a in prostate cancer. Nevertheless, the regulation and function of miR-449a in CaP remains to be better understood. miR-449a has been shown to be down-regulated in prostate cancer tissues relative to matched controls [39]. Our study is the first to demonstrate the role of miR-449a in modulating the disease phenotype of ERG fusion-positive CaP.

Many genes and different pathways have been reported in mediating the oncogenic roles of ERG. Our study, demonstrates that miR-449a acts as a tumor suppressor by inhibiting SIRT1expression, thereby triggering pathways downstream of SIRT1. SIRT1 is an NAD+-dependent histone deacetylase that regulates apoptosis in response to oxidative and genotoxic stress [40, 41]. The role of SIRT1 in tumorigenesis is controversial [42]. Recent studies indicate that SIRT1 may function as an oncogene and play a role in tumorigenesis [29, 43]. SIRT1 is overexpressed in various types of cancer including colon cancer, breast cancer, prostate cancer, squamous cell carcinoma, and human non-small cell lung cancer cell lines [43-47]. SIRT1 is significantly elevated in human prostate cancer and acts as a major epigenetic regulator [29]. Here we report that ERG induces SIRT1 expression through suppression of miR-449a. Consistently, a positive correlation between SIRT1 and ERG expression in human CaP is also observed in the analyses of the TCGA database.

SIRT1 is known to deacetylate the tumor suppressor protein p53 [48]. Therefore, we analyzed p53 expression as well as the acetylation status in ERG-associated CaP cells. Our data indicate that increased expression of ERG causes suppression of p53 expression as well as acetylation (Figure 8a). This is perhaps mediated through the miR-449a-SIRT1 axis. Consistently, we observed that suppression of SIRT1, directly by siRNA or mediated through over-expression of miR-449a restores p53 expression and also promotes increased acetylation (Figure 8b). Our findings are consistent with previous reports on the miR-449a-mediated activation of p53 pathway [49].

Our findings implicate miR-449a as a tumor suppressor in ERG-associated CaP cells by suppression of SIRT1. We found that re-introduction of miR-449a or suppression of SIRT1 inhibits the disease phenotype of CaP cells, suggesting the role of SIRT1 in regulating ERG-CaP cell migration and invasion. Our findings are consistent with other studies that have shown that SIRT1 significantly induces migration and invasion in HCC cells [50]. It has also been shown that SIRT1 induces EMT and that reduction of SIRT1 decreases prostate cancer cell migration *in vitro* [51, 52]. We find that miR-449a has antagonistic effects on the expression of SIRT1 and AR. Earlier reports indicate that SIRT1 modulate the deacetylation of the androgen receptor as well as the hormonal control of androgen receptor function [53, 54].

**Figure 9:**
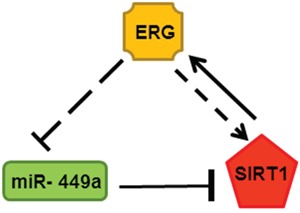
Feed-back regulatory loop associated with ERG, miR-449a (green: down-regulated) and SIRT1 (red: up-regulated) in depicted in the schematic

miR-449a targets the potential binding site within the SIRT1 3′-UTR in ERG inducible LnTE3 cells. However, miR-449a suppresses SIRT1 protein expression without affecting SIRT1 mRNA level, indicating a translational regulatory operating in these CaP cells. Moreover, we find that SIRT1 also induces ERG expression, suggesting a feedback regulatory loop. As summarized in Figure 9, increased expression of ERG results in suppression of miR-449a, and a consequential up-regulation of SIRT1, which in turn induces ERG expression.

This study underlines an important function for miR-449 in ERG-associated prostate cancer. In conclusion, this is the first report demonstrating that the suppression of the microRNA, miR-449a in ERG-positive CaP compared to ERG-negative CaP, regulates the disease phenotype. The mechanism appears to be mediated by up-regulation of SIRT1, a direct target of miR-449a. Consistently, we find that direct suppression of SIRT1 expression attenuates migration and invasion in ERG-positive CaP cells and a concurrent reduction in ERG expression. Collectively, our findings suggest an interesting feed-back regulatory loop associated with ERG, miR-449a and SIRT1. Based on earlier reports [55, 56] we considered the possibility that ERG suppresses miR-449a through transcriptional steps. *In silico* analysis indicates potential transcription factor binding sites including an ETS/ERG binding site upstream of miR-449a. Understanding the mechanisms by which ERG suppresses miR-449a and how SIRT1 regulates ERG expression are part of our future goals. These will ultimately help identify novel anti-cancer therapeutic targets for ERG-associated CaP.

## MATERIALS AND METHODS

### Human specimen

The prostate tissue specimens used in this study were obtained from patients enrolled in the Center for Prostate Disease Research (CPDR) from 1996 to 2010 under an institutional review board-approved protocol at the Walter Reed National Military Medical Center (WRNMMC) and Uniformed Services University of the Health Sciences (USUHS). Laser captured micro-dissected cells (LCM) obtained from Optimum cutting temperature (OCT) embedded tissue specimens were evaluated from benign and neoplastic epithelium of 12 CaP patients who had undergone radical prostatectomy as their primary treatment for CaP. Total RNA from the LCM derived specimens was isolated and purified [57, 58].

### Cell culture and miRNA/siRNA transfection

LnTE3 cells (LNCaP-lentivirus *TMPRESS2:ERG3*, inducible) were cultured in RPMI 1640, supplemented with 10% Tet System Approved Fetal Bovine Serum (Clontech Laboratories, Inc. Mountain View, CA, USA) and puromycin (Sigma, St. Louis, MO, USA ) with or without doxycycline (Dox, 1μg/ml) as per requirements. VCaP cell lines were cultured in DMEM supplemented with 10% fetal bovine serum (FBS). Cultures were maintained in a 5% CO2 humidified atmosphere at 37°C. For microRNA overexpression, precursor miRNA were obtained from Applied Biosystems. Cells were transfected with pre miR-449a (miR-449a, 20 nM) using siPORT NeoFX transfection reagent, whereas a scrambled miRNA precursor (miR-ctrl) was used as a negative controls. miR-874 expression was suppressed using its antagomir. Antagomir and the antagomir negative control (antagomiR-ctrl) were purchased from GenePharma Co. Ltd (Shanghai, China). The antagomir sequence (antagomiR-874) is 5′-UCGGUCCCUCGGGCCAGGGCAG-3′ and chemically modified oligonucleotides 5′-CAGUACUUUUGUGUAGUACAA-3′ were used as a negative control. Cells were transfected with the antagomir or antagomir control at 20 nM using Lipofectamine (Invitrogen, Carlsbad, CA, USA). Small interfering RNA, siSIRT1, and scrambled si-oligonucleotide were also obtained from Invitrogen. To silence SIRT1 expression, cells were transfected with siPORT NeoFX and SIRT1 antisense oligonucleotides 20 nM. Cells were harvested for protein and mRNA analysis after 48 h of transfection. For short interfering RNA (siRNA) knockdown of ERG in VCaP, the siRNA composed of the Dharmacon SMARTpool against ERG (MQ-003886-01; Lafayette, CO, USA) and non-Targeting siRNA #1 (D-001210-01) was transfected into VCaP cells as indicated using Oligofectamine (Invitrogen). After 24 hours, we carried out a second identical transfection and cells were harvested 24 hours later for RNA and protein isolation.

### Cell-based biological assays

For the invasion assay, 2.5×10^5^ LnTE3 cells were seeded into six well plate with or without doxycycline as per requirement and next day transfected with respective microRNAs or controls. After 24hr of post transfection, 1 × 10^5^ cells were suspended in serum-free medium and added into the transwell Matrigel invasion chambers (Becton Dickinson, Franklin Lakes, NJ, USA) for 48 h. To promote invasion, medium with 10% FBS as chemo-attractant were added in Bottom wells. Invaded cells on the bottom of the matrigel membrane were stained with crystal violet and then analyzed and counted under microscope. Cells in at five different areas of the membrane were counted and the experiment was repeated minimum three times. Anchorage-independent growth was determined by the Colony formation assay in soft agar. LnTE3 cells (1 × 10^5^ cells/well) were transfected and after 24 hr mixed with 2 ml of culture media containing 0.3% Difco agar Noble (Becton Dickinson, Sparks, MD, USA) and then overlaid on 1 ml of 0.5% agar in 6 well plate culture dishes. After four weeks, colonies were visualized under microscope at a magnification of 10X. Cell migration was determined using the scratch wound healing assay. For the wound scratch assay, LnTE3 cells were seeded in a 6-well culture dish with or without doxycycline and grown up to 90% confluence. A wound was made in the cell monolayer and cell debris was removed by washing twice with PBS and transfected with microRNAs or respective controls. Cells were allowed to migrate into the clearing area for 96hr. The wound closure areas were visualized under a microscope.

### Analysis and quantification of miRNA expression

For both mRNA & microRNA quantification, total RNA was isolated with the mirVana miRNA Isolation Kit (Ambion, AM1561) as per according to the manufacturer's instructions. For microRNAs analysis, RNA was reverse transcribed using the TaqMan microRNA reverse transcription kit. Each cDNA generated was then further amplified using TaqMan^®^ PreAmp Master Mix (Applied Biosytems). miRNA profiling was performed using quantitative real time-PCR utilizing TaqMan low density array (TLDA) microfluidic cards (Human miR v2.0, Applied Biosystems) on the 7500 Fast Real-Time System. qPCR data was than analyzed using StatMiner Software. Selective microRNA was validated by using TaqMan expression assay with miRNA-specific primers using 20ng RNA. RNU48 was used as an endogenous control to normalize expression. For gene expression analysis, TaqMan gene expression assay was used.

### Immunoblots and antibodies

Protein extraction and immunoblot analysis were performed using the standard protocol. Antibodies used were as follows: anti GAPDH (Millipore MAB374), Anti-ERG (Abcam ab92513), Anti-AR (Active Motif 39781), anti-PMEPA1 (ABNOVA H00056937-M01), Anti-PSA (DakoCytomation A05662), anti-p53 DO1 (Santa Cruz biotech, sc126), anti-SIRT1 and Acetyl-p53(Lys379) (Cell Signaling 2493 and 2570).

### 3′-UTR constructs/luciferase assay

LnTE3 cells were seeded at 2.5 × 10^5^ cells per well in 6-well plates. The next day, 250 ng pMIR-REPORT Luciferase vector, including 3′-UTR of SIRT1 (WT and mutant, Addgene), β-galactosidase and precursor miR-449a or scrambled controls were transfected by using siPORT NeoFX transfection reagent. Luciferase assays were performed by using the dual-light system reporter assay system (Applied Biosystems) for combined detection of firefly luciferase and β-galactosidase 48 h after transfection.

## SUPPLEMENTARY FIGURES AND TABLE





## Abbreviations

UTR: Untranslated Region
CaP: Prostate Cancer
ERG: V-Ets Avian Erythroblastosis Virus E26 Oncogene Homolog
SIRT1: Sirtuin 1
AR: Androgen Receptor
PSA: Prostate Specific Antigen
PMEPA1: Prostate Transmembrane Protein, Androgen induced 1
TMPRSS2: Transmembrane Protease Serine2
