# Supplementary material for: Loss of miR-449a in ERG-associated prostate cancer promotes the invasive phenotype by inducing SIRT1
Source: Oncotarget. 2016 Mar 14;7(16):22791–806. doi: 10.18632/oncotarget.8061 (PMC5008401; doi:10.18632/oncotarget.8061)
Supplement: Supplementary file 1 [file oncotarget-07-22791-s001.pdf]

SUPPLEMENTARY FIGURES AND TABLE

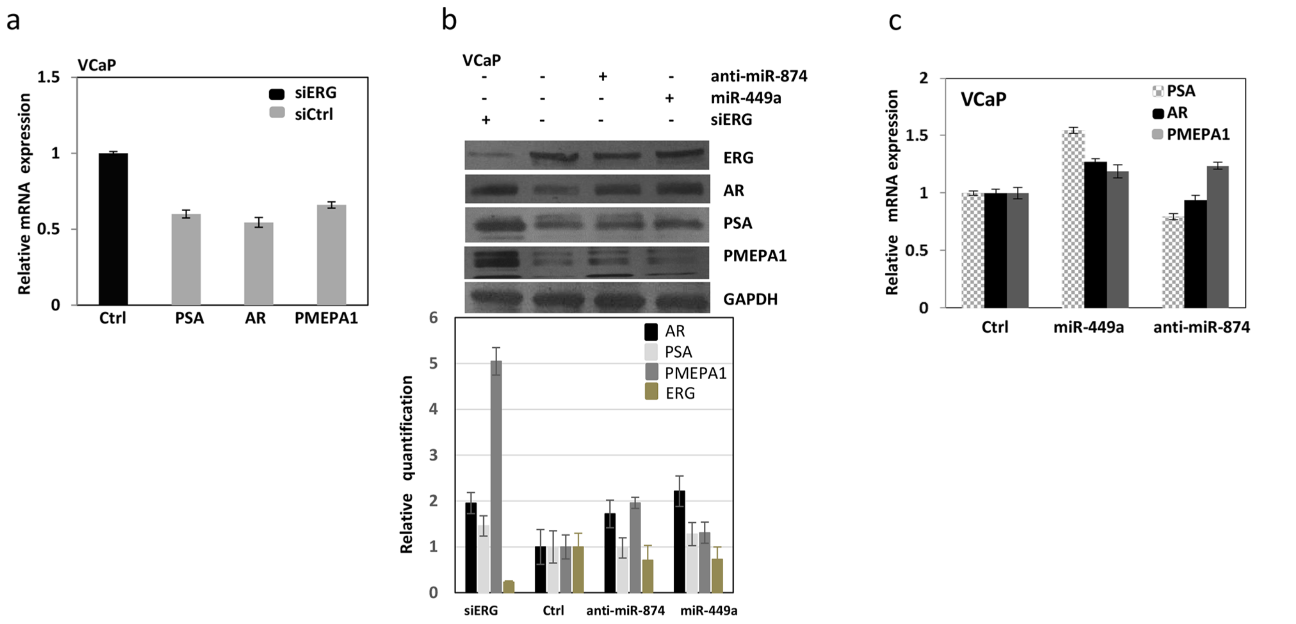

Supplementary Figure S1: AR and it target genes expression in VCaP cells. a. ERG suppressed the AR, PSA/KLK3 and PMEPA1 mRNA expression as analyzed by Real-time PCR **b.** Lanes 1 and 2, shows the AR, PSA and PMEPA1 protein expression in VCaP cells treated with ERG siRNA or control siRNA. Lanes 3 and 4, depicts the AR, PSA and PMEPA1 protein expression in VCaP cells with restoring the expression of miR-449a (by over-expression) or miR-874 (by suppression).

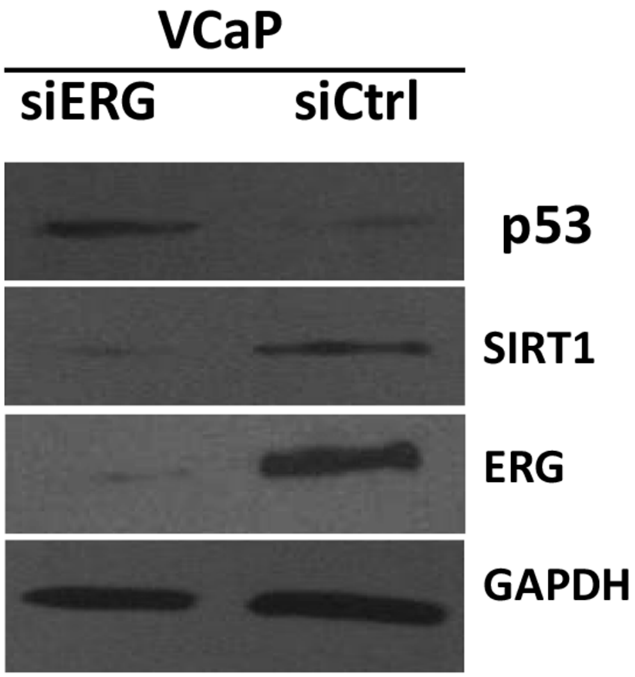

Supplementary Figure S2: ERG regulates the expression of SIRT1 and p53 in VCaP cells.

Supplementary Table S1: Comprehensive list of miRNA expressions in ERG-associated CaP, respective P values and RQ (Relative Quantitation) are indicated

See Supplementary File 1
