# Supplementary material for: Loss of miR-449a in ERG-associated prostate cancer promotes the invasive phenotype by inducing SIRT1
Source: Oncotarget. 2016 Mar 14;7(16):22791–806. doi: 10.18632/oncotarget.8061 (PMC5008401; doi:10.18632/oncotarget.8061)
Supplement: Supplementary file 2 [file oncotarget-07-22791-s002.docx]

**Supplementary Table 1:** miRNA expression levels in ERG-positive human

CaP tissues relative to ERG-negative CaP tissues

| **MicroRNAs** | **RQ_ERGpos Vs ERGneg** | **P.Value_ERGpos Vs ERGneg** |
| --- | --- | --- |
| has-miR-155-4395459 | 1.40630025 | 0.223615382 |
| hsa-let-7a-4373169 | 1.345704083 | 0.194157715 |
| hsa-let-7b-4395446 | 1.283555083 | 0.147302004 |
| hsa-let-7c-4373167 | 2.377578583 | 0.107521725 |
| hsa-let-7d-4395394 | 0.941685417 | 0.298110049 |
| hsa-let-7e-4395517 | 0.75763675 | 0.433825513 |
| hsa-let-7f-4373164 | 0.762177917 | 0.844306087 |
| hsa-let-7g-4395393 | 0.354304917 | 0.706428075 |
| hsa-miR-1-4395333 | 1.31398425 | 0.460485994 |
| hsa-miR-100-4373160 | 1.26464825 | 0.293070164 |
| hsa-miR-101-4395364 | -0.43246975 | 0.71789647 |
| hsa-miR-103-4373158 | 0.839113583 | 0.467573638 |
| hsa-miR-105-4395278 | -3.538448083 | 0.271951694 |
| hsa-miR-106a-4395280 | 0.430128417 | 0.703652737 |
| hsa-miR-106b-4373155 | 0.94996275 | 0.522921794 |
| hsa-miR-107-4373154 | -0.317188083 | 0.908772191 |
| hsa-miR-10a-4373153 | 3.261141917 | 0.112307616 |
| hsa-miR-10b-4395329 | 2.215069083 | 0.320426403 |
| hsa-miR-122-4395356 | -3.64851425 | 0.323893987 |
| hsa-miR-124-4373295 | -2.309034917 | 0.622869347 |
| hsa-miR-125a-3p-4395310 | -5.135340917 | 7.40E-03 |
| hsa-miR-125a-5p-4395309 | -0.098967083 | 0.934133878 |
| hsa-miR-125b-4373148 | 1.98702475 | 0.244552309 |
| hsa-miR-126-4395339 | 2.382094083 | 0.346549332 |
| hsa-miR-127-3p-4373147 | 1.243928083 | 0.525600278 |
| hsa-miR-127-5p-4395340 | 1.95499425 | 0.487076489 |
| hsa-miR-128-4395327 | 2.738561583 | 0.119525742 |
| hsa-miR-129-3p-4373297 | 4.52652075 | 9.95E-02 |
| hsa-miR-129-5p-4373171 | -14.25240192 | 3.45E-02 |
| hsa-miR-130a-4373145 | -3.643486583 | 0.348517827 |
| hsa-miR-130b-4373144 | 2.006772583 | 0.237950755 |
| hsa-miR-132-4373143 | 0.839941583 | 0.491958158 |
| hsa-miR-133a-4395357 | 1.457220083 | 0.451206051 |
| hsa-miR-133b-4395358 | 1.253953417 | 0.355446385 |
| hsa-miR-134-4373299 | 3.05877175 | 0.265373415 |
| hsa-miR-135a-4373140 | 0.462362417 | 0.755999186 |
| hsa-miR-135b-4395372 | -0.788251917 | 0.567102028 |
| hsa-miR-136-4373173 | 9.86E-02 | 0.958102301 |
| hsa-miR-137-4373301 | -4.31756775 | 0.44949207 |
| hsa-miR-138-4395395 | -4.922010583 | 0.296144407 |
| hsa-miR-139-3p-4395424 | 5.575815083 | 0.039967508 |
| hsa-miR-139-5p-4395400 | 1.52744675 | 0.318026485 |
| hsa-miR-140-3p-4395345 | 2.941925417 | 4.17E-02 |
| hsa-miR-140-5p-4373374 | 0.61363125 | 0.496234741 |
| hsa-miR-141-4373137 | 1.37971175 | 0.325748328 |
| hsa-miR-142-3p-4373136 | 0.730235917 | 0.555535288 |
| hsa-miR-142-5p-4395359 | -2.89677825 | 0.378975029 |
| hsa-miR-143-4395360 | 1.154256417 | 0.491845567 |
| hsa-miR-145-4395389 | 1.486757917 | 0.398592965 |
| hsa-miR-146a-4373132 | 1.019798083 | 0.536077856 |
| hsa-miR-146b-3p-4395472 | 0.478472917 | 0.822829983 |
| hsa-miR-146b-5p-4373178 | 1.313717417 | 0.398516618 |
| hsa-miR-147-4373131 | 0.36849775 | 0.852825785 |
| hsa-miR-147b-4395373 | 4.669078417 | 0.296559987 |
| hsa-miR-148a-4373130 | 0.275384583 | 0.84361257 |
| hsa-miR-148b-4373129 | 9.26E-02 | 0.933876029 |
| hsa-miR-149-4395366 | 5.294246917 | 3.48E-02 |
| hsa-miR-150-4373127 | 0.681173083 | 0.599467529 |
| hsa-miR-152-4395170 | 0.86350875 | 0.553226515 |
| hsa-miR-153-4373305 | 0.84575925 | 0.712978314 |
| hsa-miR-154-4373270 | -4.147267583 | 8.56E-02 |
| hsa-miR-15a-4373123 | 1.30010475 | 0.572508639 |
| hsa-miR-15b-4373122 | 2.668127417 | 6.84E-02 |
| hsa-miR-16-4373121 | 1.215313917 | 0.311054874 |
| hsa-miR-17-4395419 | 0.48973325 | 0.658354417 |
| hsa-miR-181a-4373117 | 2.826080583 | 0.176337695 |
| hsa-miR-181c-4373115 | 2.64745425 | 0.189255656 |
| hsa-miR-182-4395445 | 0.19466675 | 0.866416425 |
| hsa-miR-183-4395380 | 0.38100125 | 0.724279006 |
| hsa-miR-184-4373113 | 1.023910583 | 0.504756271 |
| hsa-miR-185-4395382 | 4.26116375 | 5.90E-02 |
| hsa-miR-186-4395396 | 1.15261775 | 0.304810076 |
| hsa-miR-187-4373307 | 7.727298533 | 0.354902918 |
| hsa-miR-188-3p-4395217 | 2.990026583 | 0.36697137 |
| hsa-miR-18a-4395533 | -0.86039275 | 0.581606954 |
| hsa-miR-18b-4395328 | 0.940931917 | 0.470953483 |
| hsa-miR-190-4373110 | 1.57599225 | 0.504565338 |
| hsa-miR-191-4395410 | 1.109245083 | 0.334617368 |
| hsa-miR-192-4373108 | -2.09276975 | 0.564599524 |
| hsa-miR-193a-3p-4395361 | -0.347650417 | 0.840412824 |
| hsa-miR-193a-5p-4395392 | 1.111636917 | 0.316992928 |
| hsa-miR-193b-4395478 | 1.808897417 | 0.164948099 |
| hsa-miR-194-4373106 | 0.454619083 | 0.76237585 |
| hsa-miR-195-4373105 | -3.179643917 | 0.457457544 |
| hsa-miR-196b-4395326 | 0.94576425 | 0.310605072 |
| hsa-miR-197-4373102 | -0.218605533 | 0.96558602 |
| hsa-miR-198-4395384 | 0.591099583 | 0.775074452 |
| hsa-miR-199a-3p-4395415 | 1.963727417 | 0.311802694 |
| hsa-miR-199a-5p-4373272 | -8.43E-02 | 0.984283185 |
| hsa-miR-199b-5p-4373100 | 2.728104583 | 0.299388928 |
| hsa-miR-19a-4373099 | -0.912950917 | 0.460669149 |
| hsa-miR-19b-4373098 | 0.625407583 | 0.527783124 |
| hsa-miR-200a-4378069 | -1.08886025 | 0.376981242 |
| hsa-miR-200b-4395362 | -0.40547425 | 0.673927117 |
| hsa-miR-200c-4395411 | 1.029131583 | 0.374408729 |
| hsa-miR-202-4395474 | -3.381891083 | 0.174524127 |
| hsa-miR-203-4373095 | 0.244772083 | 0.83052761 |
| hsa-miR-204-4373094 | 2.675181583 | 0.235911066 |
| hsa-miR-205-4373093 | -1.144520917 | 0.454290511 |
| hsa-miR-208-4373091 | 0.82113875 | 0.644852793 |
| hsa-miR-208b-4395401 | -1.256461583 | 0.773224784 |
| hsa-miR-20a-4373286 | 0.532771417 | 0.67344123 |
| hsa-miR-20b-4373263 | 1.24660575 | 0.255469833 |
| hsa-miR-21-4373090 | 0.424008083 | 0.71793982 |
| hsa-miR-210-4373089 | 3.105387917 | 0.096134855 |
| hsa-miR-211-4373088 | -1.86726075 | 0.614889816 |
| hsa-miR-212-4373087 | -0.58630125 | 0.664826735 |
| hsa-miR-214-4395417 | 3.17245175 | 0.135873399 |
| hsa-miR-215-4373084 | 1.751671417 | 0.293999621 |
| hsa-miR-216a-4395331 | -1.869754417 | 0.569696711 |
| hsa-miR-216b-4395437 | 1.425192917 | 0.657518818 |
| hsa-miR-217-4395448 | 3.05038375 | 0.52367374 |
| hsa-miR-218-4373081 | 1.942887583 | 0.166599736 |
| hsa-miR-219-1-3p-4395206 | -0.80983275 | 0.701654248 |
| hsa-miR-219-2-3p-4395501 | 0.21348375 | 0.908028264 |
| hsa-miR-219-5p-4373080 | -0.448052083 | 0.899697616 |
| hsa-miR-22-4373079 | 5.559987083 | 0.129611372 |
| hsa-miR-220-4373078 | 4.214922583 | 0.126505332 |
| hsa-miR-220b-4395317 | -1.85389875 | 0.572796924 |
| hsa-miR-220c-4395322 | -9.840743917 | 5.37E-02 |
| hsa-miR-221-4373077 | 2.83749375 | 0.152912697 |
| hsa-miR-222-4395387 | 1.64402125 | 0.41615811 |
| hsa-miR-223-4395406 | 1.110896083 | 0.528685731 |
| hsa-miR-224-4395210 | -4.669533367 | 0.513411977 |
| hsa-miR-23a-4373074 | -7.3411185 | 0.225066611 |
| hsa-miR-23b-4373073 | 3.050799583 | 0.229877421 |
| hsa-miR-24-4373072 | 1.22143125 | 0.41743252 |
| hsa-miR-25-4373071 | 0.52803275 | 0.609336795 |
| hsa-miR-26a-4395166 | 0.809314417 | 0.572006533 |
| hsa-miR-26b-4395167 | 0.333227083 | 0.748436613 |
| hsa-miR-27a-4373287 | -1.189722583 | 0.680754001 |
| hsa-miR-27b-4373068 | 1.598206417 | 0.329445301 |
| hsa-miR-28-3p-4395557 | 1.110344083 | 0.24055008 |
| hsa-miR-28-5p-4373067 | 1.12621175 | 0.313466996 |
| hsa-miR-296-3p-4395212 | -6.392707917 | 0.212722244 |
| hsa-miR-296-5p-4373066 | 0.567396083 | 0.745653788 |
| hsa-miR-298-4395301 | -0.62367375 | 0.660093381 |
| hsa-miR-299-3p-4373189 | 5.769153583 | 0.255862562 |
| hsa-miR-299-5p-4373188 | 1.537367417 | 0.577146815 |
| hsa-miR-29a-4395223 | 1.518106917 | 0.306536502 |
| hsa-miR-29b-4373288 | 1.370302417 | 0.488546767 |
| hsa-miR-29c-4395171 | 1.119466917 | 0.393314596 |
| hsa-miR-301a-4373064 | 0.70435225 | 0.513245452 |
| hsa-miR-301b-4395503 | 0.826528417 | 0.670856032 |
| hsa-miR-302a-4378070 | 0.729884917 | 0.715938514 |
| hsa-miR-302b-4378071 | -1.016865083 | 0.562704606 |
| hsa-miR-302c-4378072 | 5.64E-02 | 0.984378925 |
| hsa-miR-30b-4373290 | 0.902927417 | 0.435476496 |
| hsa-miR-30c-4373060 | 0.770923417 | 0.518733569 |
| hsa-miR-31-4395390 | -5.00E-02 | 0.981494197 |
| hsa-miR-32-4395220 | 0.97284825 | 0.471000739 |
| hsa-miR-320-4395388 | 1.95117425 | 0.10802404 |
| hsa-miR-323-3p-4395338 | -1.972875417 | 0.14448387 |
| hsa-miR-324-3p-4395272 | 1.484242917 | 0.222661618 |
| hsa-miR-324-5p-4373052 | 2.180388583 | 0.158346131 |
| hsa-miR-325-4373051 | -2.50014225 | 0.598577127 |
| hsa-miR-326-4373050 | -0.889623083 | 0.596339455 |
| hsa-miR-328-4373049 | 1.277733583 | 0.382674042 |
| hsa-miR-329-4373191 | -0.592914583 | 0.665359069 |
| hsa-miR-330-3p-4373047 | 3.732911083 | 8.72E-02 |
| hsa-miR-330-5p-4395341 | 5.593428583 | 0.25609389 |
| hsa-miR-331-3p-4373046 | 2.007062917 | 0.218386558 |
| hsa-miR-331-5p-4395344 | 2.784352583 | 0.028219913 |
| hsa-miR-335-4373045 | 2.950406917 | 0.210830782 |
| hsa-miR-337-5p-4395267 | 1.260020083 | 0.684768385 |
| hsa-miR-338-3p-4395363 | -7.91199375 | 0.124300574 |
| hsa-miR-339-3p-4395295 | 1.507707417 | 0.15651423 |
| hsa-miR-339-5p-4395368 | -6.589527583 | 0.123496981 |
| hsa-miR-33b-4395196 | 1.247632917 | 0.631388739 |
| hsa-miR-340-4395369 | -0.410322417 | 0.74655208 |
| hsa-miR-342-3p-4395371 | 1.070416083 | 0.362424656 |
| hsa-miR-342-5p-4395258 | 4.51610075 | 8.32E-02 |
| hsa-miR-345-4395297 | 1.114335083 | 0.508580237 |
| hsa-miR-346-4373038 | 4.071753917 | 0.216235302 |
| hsa-miR-34a-4395168 | 0.28295325 | 0.82116537 |
| hsa-miR-34c-5p-4373036 | 3.192025417 | 0.238469384 |
| hsa-miR-361-5p-4373035 | 1.29513125 | 0.567062404 |
| hsa-miR-362-3p-4395228 | 2.052808917 | 0.181285855 |
| hsa-miR-362-5p-4378092 | 3.50528725 | 4.09E-02 |
| hsa-miR-363-4378090 | -4.50E-02 | 0.966642872 |
| hsa-miR-365-4373194 | 1.094007917 | 0.265311192 |
| hsa-miR-367-4373034 | 1.80500175 | 0.462611055 |
| hsa-miR-369-3p-4373032 | -0.38983065 | 0.947012281 |
| hsa-miR-369-5p-4373195 | -3.25658525 | 0.660660908 |
| hsa-miR-370-4395386 | 7.12185325 | 1.86E-02 |
| hsa-miR-371-3p-4395235 | -0.718045083 | 0.931897043 |
| hsa-miR-372-4373029 | 0.310523083 | 0.866268889 |
| hsa-miR-373-4378073 | -0.321125583 | 0.878811386 |
| hsa-miR-374a-4373028 | 0.281066083 | 0.787976318 |
| hsa-miR-374b-4381045 | 1.101411583 | 0.426398344 |
| hsa-miR-375-4373027 | 1.105357917 | 0.223819285 |
| hsa-miR-376a-4373026 | -1.39714275 | 0.661552164 |
| hsa-miR-376b-4373196 | 6.488012583 | 0.377460738 |
| hsa-miR-376c-4395233 | 2.159320083 | 0.330786699 |
| hsa-miR-377-4373025 | 3.807165417 | 0.223550727 |
| hsa-miR-379-4373349 | 2.63690175 | 0.271774398 |
| hsa-miR-380-4373022 | 1.93810225 | 0.752787081 |
| hsa-miR-381-4373020 | -1.73972675 | 0.480859248 |
| hsa-miR-382-4373019 | 7.71421125 | 2.93E-02 |
| hsa-miR-383-4373018 | -3.914692917 | 0.260578269 |
| hsa-miR-384-4373017 | -1.002061917 | 0.780828949 |
| hsa-miR-409-5p-4395442 | 1.103781083 | 0.666462877 |
| hsa-miR-410-4378093 | -0.87270425 | 0.618114011 |
| hsa-miR-411-4381013 | 1.981423583 | 0.383212057 |
| hsa-miR-412-4373199 | 2.879807417 | 0.200780125 |
| hsa-miR-422a-4395408 | 0.86702675 | 0.802393581 |
| hsa-miR-423-5p-4395451 | 3.00320675 | 8.59E-02 |
| hsa-miR-424-4373201 | 1.513485417 | 0.735491085 |
| hsa-miR-425-4380926 | -0.519248417 | 0.65174572 |
| hsa-miR-429-4373203 | 0.17324525 | 0.893551479 |
| hsa-miR-431-4395173 | -1.17189975 | 0.366272922 |
| hsa-miR-433-4373205 | 6.14347425 | 0.171712585 |
| hsa-miR-448-4373206 | -0.62367375 | 0.660093381 |
| hsa-miR-449a-4373207 | 7.74792125 | 1.22E-03 |
| hsa-miR-449b-4381011 | 4.745000083 | 0.12759085 |
| hsa-miR-450a-4395414 | -0.373097583 | 0.852616605 |
| hsa-miR-450b-3p-4395319 | -7.5730652 | 0.236764018 |
| hsa-miR-450b-5p-4395318 | -0.18523175 | 0.964565868 |
| hsa-miR-451-4373360 | 1.837356583 | 0.299126884 |
| hsa-miR-452-4395440 | 0.428131417 | 0.85265119 |
| hsa-miR-453-4395429 | -0.62367375 | 0.660093381 |
| hsa-miR-454-4395434 | -1.697057917 | 0.533349292 |
| hsa-miR-455-3p-4395355 | 1.628103917 | 0.447641505 |
| hsa-miR-455-5p-4378098 | 0.799939083 | 0.718625577 |
| hsa-miR-483-5p-4395449 | 1.426182917 | 0.503436794 |
| hsa-miR-484-4381032 | 2.11038675 | 0.174840582 |
| hsa-miR-485-3p-4378095 | -3.325741917 | 0.060547265 |
| hsa-miR-485-5p-4373212 | -0.953040083 | 0.842019916 |
| hsa-miR-486-3p-4395204 | 1.80028025 | 0.561334177 |
| hsa-miR-486-5p-4378096 | -0.21045425 | 0.929655292 |
| hsa-miR-487a-4378097 | 0.993666917 | 0.809921839 |
| hsa-miR-487b-4378102 | -1.971087417 | 0.360258711 |
| hsa-miR-488-4395468 | -0.62367375 | 0.660093381 |
| hsa-miR-489-4395469 | 1.206814583 | 0.650103039 |
| hsa-miR-490-3p-4373215 | -2.353358917 | 0.448908083 |
| hsa-miR-491-3p-4395471 | 0.616005583 | 0.775974609 |
| hsa-miR-491-5p-4381053 | -0.125904583 | 0.937052156 |
| hsa-miR-492-4373217 | 2.90677025 | 0.191322741 |
| hsa-miR-493-4395475 | -6.443879917 | 0.2266718 |
| hsa-miR-494-4395476 | 1.276586583 | 0.41454156 |
| hsa-miR-495-4381078 | 0.29545975 | 0.864306257 |
| hsa-miR-496-4386771 | 1.419069083 | 0.666957217 |
| hsa-miR-499-3p-4395538 | 1.36379475 | 0.660004062 |
| hsa-miR-499-5p-4381047 | 1.702767917 | 0.734324606 |
| hsa-miR-500-4395539 | 2.282226083 | 0.448305112 |
| hsa-miR-501-3p-4395546 | -0.812510917 | 0.768773997 |
| hsa-miR-501-5p-4373226 | 4.21402325 | 0.138103685 |
| hsa-miR-502-3p-4395194 | 2.883211917 | 6.64E-02 |
| hsa-miR-502-5p-4373227 | -1.668764583 | 0.411290544 |
| hsa-miR-503-4373228 | -5.589887083 | 0.292881474 |
| hsa-miR-504-4395195 | 2.595911583 | 0.191761422 |
| hsa-miR-505-4395200 | -7.90699625 | 0.117386221 |
| hsa-miR-506-4373231 | -2.176296583 | 0.32034367 |
| hsa-miR-507-4373232 | 8.573879167 | 0.142123216 |
| hsa-miR-508-3p-4373233 | -7.09385675 | 0.121816227 |
| hsa-miR-508-5p-4395203 | 2.821345417 | 0.403449824 |
| hsa-miR-509-3-5p-4395266 | -5.713692417 | 0.310796164 |
| hsa-miR-509-5p-4395346 | 9.79E-02 | 0.975524569 |
| hsa-miR-510-4395352 | -0.62367375 | 0.660093381 |
| hsa-miR-511-4373236 | -4.75E-02 | 0.985221946 |
| hsa-miR-512-3p-4381034 | 5.457655417 | 0.337455173 |
| hsa-miR-512-5p-4373238 | -4.885039083 | 0.347463686 |
| hsa-miR-513-5p-4395201 | -0.62367375 | 0.660093381 |
| hsa-miR-515-3p-4395480 | 6.393823167 | 0.420419635 |
| hsa-miR-515-5p-4373242 | 1.50295725 | 0.645414473 |
| hsa-miR-516a-5p-4395527 | 1.747469583 | 0.566899963 |
| hsa-miR-516b-4395172 | -2.000719417 | 0.281417671 |
| hsa-miR-517a-4395513 | 2.069819483 | 0.779583815 |
| hsa-miR-517b-4373244 | 1.847728917 | 0.57904316 |
| hsa-miR-517c-4373264 | 0.22200215 | 0.9786651 |
| hsa-miR-518a-3p-4395508 | -4.87387775 | 0.523220255 |
| hsa-miR-518a-5p-4395507 | 4.496867583 | 0.416626103 |
| hsa-miR-518b-4373246 | 10.82349145 | 8.51E-02 |
| hsa-miR-518c-4395512 | 0.3966103 | 0.953735463 |
| hsa-miR-518d-3p-4373248 | -1.46106275 | 0.6320827 |
| hsa-miR-518d-5p-4395500 | -1.194944583 | 0.865151529 |
| hsa-miR-518e-4395506 | -0.360081917 | 0.947144662 |
| hsa-miR-518f-4395499 | 4.11596425 | 0.316713233 |
| hsa-miR-519a-4395526 | -3.847758533 | 0.422416729 |
| hsa-miR-519c-3p-4373251 | -5.8031388 | 0.30100646 |
| hsa-miR-519d-4395514 | 9.714139483 | 0.173494963 |
| hsa-miR-519e-4395481 | 0.939991417 | 0.717813201 |
| hsa-miR-520a-3p-4373268 | -12.00277908 | 8.68E-02 |
| hsa-miR-520a-5p-4378085 | 3.73249925 | 0.380855226 |
| hsa-miR-520b-4373252 | -1.065536583 | 0.722035923 |
| hsa-miR-520d-5p-4395504 | 1.225343917 | 0.537002348 |
| hsa-miR-520e-4373255 | 4.740474583 | 0.18265447 |
| hsa-miR-520f-4373256 | 4.31161375 | 2.46E-02 |
| hsa-miR-520g-4373257 | -21.76229598 | 2.75E-03 |
| hsa-miR-521-4373259 | 4.167141633 | 0.528201536 |
| hsa-miR-522-4395524 | -2.354650433 | 0.748933081 |
| hsa-miR-523-4395497 | -0.61748375 | 0.71795179 |
| hsa-miR-524-5p-4395174 | -0.62367375 | 0.660093381 |
| hsa-miR-525-3p-4395496 | 4.171084083 | 0.390870091 |
| hsa-miR-525-5p-4378088 | -8.034321683 | 0.12443834 |
| hsa-miR-526b-4395493 | -3.55664675 | 0.54036615 |
| hsa-miR-532-3p-4395466 | 2.158552917 | 6.78E-02 |
| hsa-miR-532-5p-4380928 | 6.497636917 | 1.50E-02 |
| hsa-miR-539-4378103 | 10.61794368 | 4.21E-02 |
| hsa-miR-541-4395312 | 10.47752118 | 0.176544494 |
| hsa-miR-542-3p-4378101 | 2.499112417 | 0.56663656 |
| hsa-miR-542-5p-4395351 | 5.03684825 | 0.458330225 |
| hsa-miR-544-4395376 | 2.581634917 | 0.254549151 |
| hsa-miR-545-4395378 | 1.002734917 | 0.581917955 |
| hsa-miR-548a-3p-4380948 | -1.19995725 | 0.524386431 |
| hsa-miR-548a-5p-4395523 | 4.282891583 | 0.496234007 |
| hsa-miR-548b-3p-4380951 | 2.565367917 | 0.446032998 |
| hsa-miR-548b-5p-4395519 | 0.27123325 | 0.894956592 |
| hsa-miR-548c-3p-4380993 | -0.95134775 | 0.653494983 |
| hsa-miR-548c-5p-4395540 | -1.936156583 | 0.194431219 |
| hsa-miR-548d-3p-4381008 | 6.661219083 | 0.429735632 |
| hsa-miR-548d-5p-4395348 | -0.69024325 | 0.728532075 |
| hsa-miR-551b-4380945 | 0.379771917 | 0.910627405 |
| hsa-miR-556-3p-4395456 | -2.768764417 | 0.158269445 |
| hsa-miR-556-5p-4395455 | 0.49358525 | 0.877529216 |
| hsa-miR-561-4380938 | 1.957086083 | 0.712040004 |
| hsa-miR-570-4395458 | -1.098288417 | 0.570954269 |
| hsa-miR-574-3p-4395460 | 1.472641583 | 0.189393973 |
| hsa-miR-576-3p-4395462 | 7.170457917 | 4.47E-02 |
| hsa-miR-576-5p-4395461 | 0.721991583 | 0.87427965 |
| hsa-miR-579-4395509 | 1.427490083 | 0.450066582 |
| hsa-miR-582-3p-4395510 | -2.352372917 | 0.155783941 |
| hsa-miR-582-5p-4395175 | 0.519945917 | 0.841668714 |
| hsa-miR-589-4395520 | 5.229351583 | 2.23E-03 |
| hsa-miR-590-5p-4395176 | 1.56273125 | 0.292346553 |
| hsa-miR-597-4380960 | -0.04627475 | 0.978379374 |
| hsa-miR-598-4395179 | 0.610203583 | 0.514112824 |
| hsa-miR-615-3p-4386777 | 2.378503917 | 0.335299248 |
| hsa-miR-615-5p-4395464 | 2.817535583 | 0.120009832 |
| hsa-miR-616-4395525 | -1.544495417 | 0.757775996 |
| hsa-miR-618-4380996 | 1.43865075 | 0.791174777 |
| hsa-miR-624-4395541 | 4.204629917 | 0.174147874 |
| hsa-miR-625-4395542 | 1.135087083 | 0.47922794 |
| hsa-miR-627-4380967 | -0.549157783 | 0.836720974 |
| hsa-miR-628-5p-4395544 | -0.761653 | 0.811050953 |
| hsa-miR-629-4395547 | 2.83214675 | 0.061058074 |
| hsa-miR-636-4395199 | 1.785040083 | 0.438009017 |
| hsa-miR-642-4380995 | -1.018142917 | 0.681332079 |
| hsa-miR-651-4381007 | 1.423283917 | 0.608066621 |
| hsa-miR-652-4395463 | 1.77168375 | 0.243801741 |
| hsa-miR-653-4395403 | -7.812664583 | 0.066867488 |
| hsa-miR-654-3p-4395350 | -5.12535025 | 6.56E-02 |
| hsa-miR-654-5p-4381014 | -0.87508675 | 0.612763413 |
| hsa-miR-655-4381015 | -1.052450083 | 0.595525137 |
| hsa-miR-660-4380925 | 4.881589917 | 4.58E-02 |
| hsa-miR-671-3p-4395433 | 1.487314583 | 0.166475456 |
| hsa-miR-672-4395438 | -3.612555083 | 0.515716439 |
| hsa-miR-674-4395193 | -11.28192992 | 5.47E-02 |
| hsa-miR-708-4395452 | 8.95E-02 | 0.931136501 |
| hsa-miR-744-4395435 | 1.816850083 | 0.146860522 |
| hsa-miR-758-4395180 | 1.84518075 | 0.32807126 |
| hsa-miR-871-4395465 | -5.425892083 | 0.358377213 |
| hsa-miR-872-4395375 | 12.67713158 | 4.29E-02 |
| hsa-miR-873-4395467 | 2.9527883 | 0.651467673 |
| hsa-miR-874-4395379 | -22.65634258 | 1.32E-03 |
| hsa-miR-875-3p-4395315 | 2.702952417 | 0.56619119 |
| hsa-miR-876-3p-4395336 | 4.076958917 | 0.591926232 |
| hsa-miR-876-5p-4395316 | 3.04E-02 | 0.986991653 |
| hsa-miR-885-3p-4395483 | -3.49151325 | 0.272324817 |
| hsa-miR-885-5p-4395407 | 3.072220417 | 0.455452637 |
| hsa-miR-886-3p-4395305 | 4.023208083 | 8.27E-02 |
| hsa-miR-886-5p-4395304 | 3.703729417 | 2.71E-02 |
| hsa-miR-887-4395485 | -3.866005917 | 0.432826258 |
| hsa-miR-888-4395323 | 1.02167125 | 0.798671584 |
| hsa-miR-889-4395313 | -6.97880925 | 0.167823603 |
| hsa-miR-890-4395320 | 7.835360217 | 0.258388988 |
| hsa-miR-891a-4395302 | -1.065683083 | 0.536868763 |
| hsa-miR-891b-4395321 | 6.919344917 | 0.197057845 |
| hsa-miR-892a-4395306 | 0.49294925 | 0.7288966 |
| hsa-miR-9-4373285 | 2.708761083 | 0.309685125 |
| hsa-miR-92a-4395169 | 0.224619583 | 0.814118894 |
| hsa-miR-93-4373302 | 0.751344417 | 0.454591656 |
| hsa-miR-95-4373011 | 2.179374417 | 0.232678108 |
| hsa-miR-96-4373372 | 2.21567975 | 0.190498156 |
| hsa-miR-98-4373009 | -2.797636417 | 0.379222707 |
| hsa-miR-99a-4373008 | 2.539057083 | 0.056366086 |
| hsa-miR-99b-4373007 | 1.092489583 | 0.490450315 |
